# Head Start Immunity: Characterizing the Early Protection of C Strain Vaccine Against Subsequent Classical Swine Fever Virus Infection

**DOI:** 10.3389/fimmu.2019.01584

**Published:** 2019-07-23

**Authors:** Ronan R. McCarthy, Helen E. Everett, Simon P. Graham, Falko Steinbach, Helen R. Crooke

**Affiliations:** ^1^Virology Department, Animal and Plant Health Agency, Addlestone, United Kingdom; ^2^School of Veterinary Medicine, University of Surrey, Guildford, United Kingdom; ^3^The Pirbright Institute, Pirbright, United Kingdom

**Keywords:** swine, CSFV, innate, C Strain, ISG15, antiviral, vaccination

## Abstract

Classical Swine Fever Virus (CSFV) is an ongoing threat to the pig industry due to the high transmission and mortality rates associated with infection. Live attenuated vaccines such as the CSFV C strain vaccine are capable of protecting against infection within 5 days of vaccination, but the molecular mechanisms through which this early protection is mediated have yet to be established. In this study, we compared the response of pigs vaccinated with the C strain to non-vaccinated pigs both challenged with a pathogenic strain of CSFV. Analysis of transcriptomic data from the tonsils of these animals during the early stages after vaccination and challenge reveals a set of regulated genes that appear throughout the analysis. Many of these are linked to the ISG15 antiviral pathway suggesting it may play a role in the rapid and early protection conferred by C strain vaccination.

## Introduction

Classical Swine Fever (CSF) is a contagious, haemorrhagic and often fatal disease of suidae such as pigs and wild boar, caused by the classical swine fever virus (CSFV). CSFV is an enveloped, single-stranded RNA virus that belongs to the pestivirus genus of the *Flaviviridae* family (1). The positive-sense RNA genome of ~12.3 kb is translated as a single polyprotein that is then cleaved by both host and native proteases to form 11 proteins, 4 of which are structural components of the virion (2). Of these structural proteins 2 envelope glycoproteins, E1 and E2, are required for virus entry into the cell through clathrin-dependent, receptor-mediated endocytosis (3). The primary site of replication are the tonsils and oropharyngeal lymph nodes. From here, the virus is transported through the lymphatic system to the primary lymph nodes, where further rounds of replication occur until the virus eventually reaches all other organs in the body via the circulatory system (4). Interferon signaling is a key component of how the innate immune system responds to challenge with CSFV. High levels of interferon-α (IFN- α) are a characteristic feature of acute disease (5). The levels of induction are associated with the virulence of the strain, with highly virulent strains inducing the highest levels (6–8). Despite the classical functional role of IFNs during viral infection, which is to induce the expression of a cohort of antiviral proteins, these high levels of IFN-α are counterproductive. They do not limit virus replication, and lead to the development of disease-associated immunopathology observed through severe lymphoid depletion, lymphocyte apoptosis and thrombocytopenia. This immune dysfunction presents clinically as a viral haemorrhagic fever (5).

CSF is endemic to parts of South East Asia, Russia and South America. Within Europe, stringent controls such as a stamping out policy, movement restrictions and epidemiological surveillance measures have been in place since 1990 to prevent the spread of the disease, however, sporadic outbreaks have occurred, for example in Lithuania and Latvia, and the recent reoccurrence of CSF in Japan after 26 year absence highlights that CSFV remains an epizootic threat (9, 10). CSF is amenable to control by vaccination with a number of different live attenuated vaccines available, the most widely used of which is the C strain vaccine (9). However, the inability to distinguish serologically between animals that have been vaccinated or are infected with the virus (DIVA) means its use as an outbreak control tool is limited in CSF-free countries (2). The C strain vaccine was generated through serial passage in rabbits until it was no longer pathogenic. It provides a rapid and complete protection of pigs against infection and also prevents viral transmission within 5 days of vaccination (11, 12). The immunological signaling cascades behind the early protection afforded by C strain are poorly understood, but precede the adaptive response, where IFNγ^+^ CD8^+^ cells precede the detection of a humoral, virus neutralizing response (13–15). As the C strain vaccine has been the most widely used vaccine for CSFV to date, deciphering the precise innate immune signaling pathways underpinning its effectiveness may help shape and optimize the current generation of marker and subunit vaccines. To achieve a greater insight into the host response to vaccination with C strain, porcine microarrays were utilized to analyse the differences in gene expression in tonsil tissue between pigs that were vaccinated with C strain or given a mock inoculum. These pigs were then subsequently challenged with a virulent strain of CSFV five days post immunization, thus before an effective adaptive response could be mounted. In this study we have examined transcriptional changes in tonsils at early time points to identify subsets of genes that may be integral to this rapid protection and could support the induction of an early adaptive immune response.

## Materials and Methods

### Viruses

C strain CSFV (AC Riemser Schweinepestvakzine, Riemser Arzneimittel AG, Riems, Germany) and the virulent CSFV Brescia strain were propagated in PK15 cell monolayers. Both mock virus and virus stocks were prepared, and titers were determined, as described previously for this animal cohort (15).

### Ethics Statement

All animal work was approved by the Animal and Plant Health Agency (APHA) Animal Welfare and Ethical Review Board, and all procedures were conducted in accordance with the Animals (Scientific Procedures) Act 1986 (United Kingdom) under project license permit PPL 70/6559. Each animal was euthanized on predetermined days by stunning and exsanguination.

### Animals

Eighteen Large White/Landrace crossbreed pigs of 9 weeks of age were randomly assigned to one of two groups. On day 0 the animals in group 1 (*n* = 9) were vaccinated with 2 ml of C strain vaccine into the brachiocephalous muscle (as recommended by the manufacturer), and group 2 (*n* = 9) was intranasally inoculated with tissue culture supernatant (mock). For intranasal inoculations 1 ml per nostril was administered using a mucosal atomization device (MAD300; Wolf Tory Medical, USA). On day 5 post vaccination (dpv), both groups were inoculated intranasally with 10^5^ TCID_50_ of CSFV Brescia strain. EDTA anti-coagulated blood samples were collected in Vacutainers (BD Biosciences), prior to and after challenge, from the external jugular vein. Three animals from each group were euthanized on dpv 5 (prior to challenge), dpv 8 and dpv10 and the tonsils were collected.

### Clinical, Hematological, and Virological Methods

The animals were inspected by the APHA Animal Sciences Unit staff twice daily (am and pm), and 10 parameters relevant to an indication of CSF (temperature, liveliness, body shape and tension, breathing, walking, skin, eye/conjunctiva, appetite, and defecation) were examined and scored as 0 (normal), to 3 (severely altered; known CSF sign) (16). A total clinical score for each animal was assigned twice daily, and their temperatures were monitored by rectal thermometer readings once daily. Peripheral blood leukocytes and CSFV RNA were monitored in EDTA blood samples collected every 3 days using volumetric flow cytometry and real-time reverse transcription-quantitative RT-PCR (RRT-qPCR), respectively (16).

### Gene Expression Microarray Analysis

At days 5, 8, and 10 post-vaccination animals were euthanized, the tonsils removed, chopped into fine pieces and stored at −80°C in RNAlater (Sigma-Aldrich). RNA was extracted using MagMax 96 microarray total RNA isolation kit which includes a Turbo DNAse treatment to remove contaminating genomic DNA. Elimination of genomic DNA was confirmed by q-PCR detection of porcine β*-actin* gene with and without reverse transcription. The Ovation PicoSL WTA System v2 kit (NuGEN, Leek, The Netherlands) was used to amplify cDNA from 50 ng total RNA. The MinElute Reaction Cleanup Kit (Qiagen) was used to purify cDNA, and 1 μg was then labeled using a one-color DNA labeling kit (NimbleGen, Madison, USA). For each sample, 4 μg labeled cDNA was hybridized to a custom NimbleGen 12 × 135 K porcine array designed using the *Sus scrofa* 10.2 genome build and incorporating a total of 19,351 genes, each represented on the array by a set of six different probes (116,106 probes in total) (17). The microarray also contained a large number (24,179) of random probes. Hybridized arrays were scanned at 2 μm resolution on a microarray scanner (Agilent, Wokingham, UK). Microarray images were processed using DEVA v1.2.1 software to obtain a pair report containing the signal intensity values for each probe. To correct for differences in the overall intensity levels between slides robust multi-array (RMA) normalization was used. Data was then processed using GeneSpring GX using the manufacturer's guidelines. RMA normalized pair files were imported and empirical Bayesian unpaired comparison (moderated *t*-test, *P* < 0.05), combined with a Westfall and Young Permutation to correct for multiple testing, was carried out to generate a list of genes with significantly altered expression, between C strain and mock inoculated pigs, of > 2-fold. The raw microarray data (background-corrected signal) can be assessed at Gene Expression Omnibus (GEO accession GSE111486).

### Gene Ontology and Pathway Analysis

To aid in the analysis of the data, where possible human ortholog of porcine genes were used for further analysis. Gene Ontology analysis was performed using BiNGO within Cytoscape 3.2 (18, 19). BiNGO analysis was performed using a hypergeometric test with a Benjamini Hochberg False Discovery Rate correction and significance value of 0.05, the ontology file used was GO_Biological_Process. PANTHER Overrepresentation Analysis (release 20171205) was performed using the annotation Reactome version 58 (Release 20161207) using a Binomial test with a Bonferroni Correction for multiple comparison (20, 21). Network analysis was performed using NetworkAnalyzer tool in Cytoscape, nodes and label sizes are mapped based on betweenness centrality and isolated nodes (degree = 0) removed (18).

## Results

### Vaccination, Challenge and Clinical Observations

**Figure 1:**
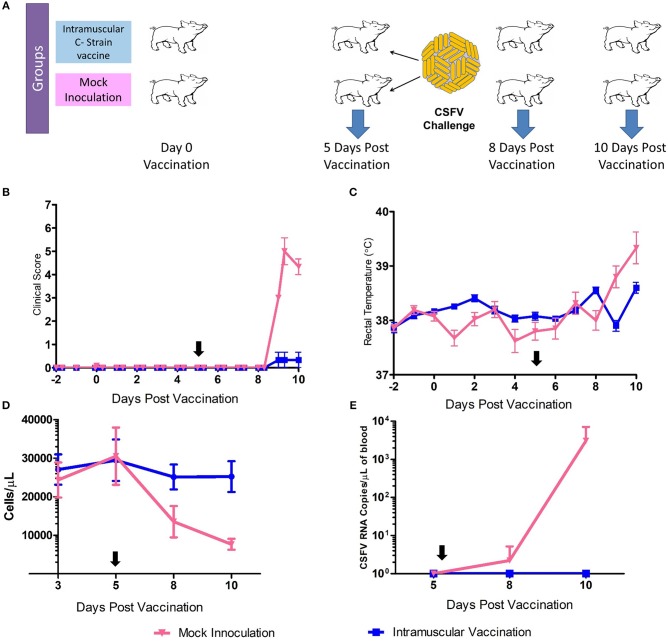
C Strain Vaccination and Subsequent Challenge. **(A)** Schematic outline of the vaccine/challenge study highlighting the key time points of vaccination and challenge. Three animals per group were euthanized at day 5, 8, and 10 pv for sample acquisition (Blue arrows). **(B)** Mean clinical score data from both C strain vaccinated and mock inoculated animals from before the study commenced until completion. **(C)** Rectal temperatures of animals throughout the course of the study. **(D)** Peripheral blood leukocyte counts in EDTA blood samples throughout the study **(E)** CSFV RNA as detected in blood by reverse transcription-quantitative RT-PCR. Black arrows indicate time of challenge. Error bars indicate SD.

Samples for transcriptomic analysis were generated from animals vaccinated, or mock inoculated, 5 days prior to challenge with virulent CSFV (15). Tonsil samples were collected prior to challenge at 5 dpv and also at day 8 and day 10 pv [3 and 5 days post-challenge (dpc)] (Figure 1A). C strain vaccinated animals were protected from the challenge with no clinical signs or temperature increase detected. The mock inoculated animals had early clinical signs of CSF (inappetence, lethargy and reddening of conjunctiva) from 4 to 5 dpc and elevated rectal temperatures (Figures 1B,C). CSFV RNA and leukopenia was detected in blood samples from 8 dpv in the mock inoculated animals but not in vaccinated animals (Figures 1D,E). This level of protection corresponds with previous studies in that complete protection from challenge with CSFV was observed within 5 days of vaccination, thus before the onset of an adaptive immune response, which then rapidly develops after challenge (12).

### Intramuscular Vaccination Produces a Robust Transcriptional Response in Tonsil Cells of Naïve Pigs

**Figure 2:**
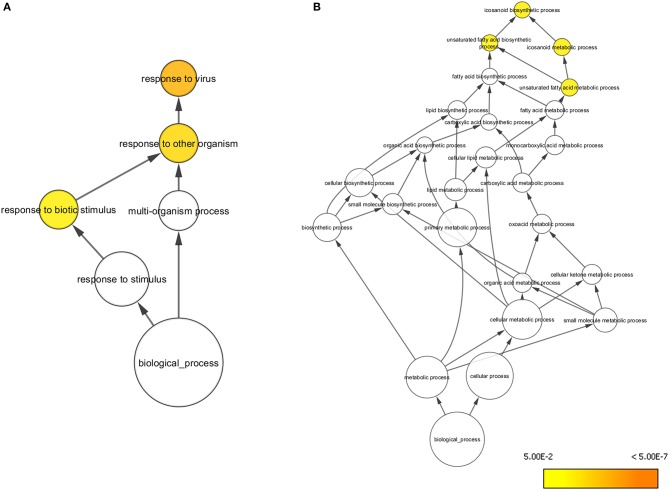
Overrepresented Gene Ontologies in tonsils 5 days after vaccination. **(A)** Gene ontologies overrepresented in the subset of genes upregulated in C strain vaccinated pigs at 5 days post vaccination. **(B)** Gene ontologies overrepresented in the subset of genes downregulated in C strain vaccinated pigs at 5 days post vaccination. Hypergeometric Test used to determine significance (*p* < 0.05). Level of significance indicated by yellow to orange coloring.

At day 5 post-vaccination (prior to challenge), when vaccinated pigs were compared to mock inoculated pigs, 448 genes were differentially regulated; 255 genes were down-regulated and 193 genes upregulated (Table 1, Supplementary Table 1). Gene Ontology analysis (19) highlighted over representation of gene categories associated with response to virus among the upregulated genes as expected since the C strain vaccine is a live, attenuated virus (Figure 2A). Among the downregulated genes a number of different metabolic processes were over-represented which could suggest an appropriation of cellular processes and resources toward the production of antiviral effectors (Figure 2B).

**Table 1:** Genes differentially regulated in C strain vaccinated pigs compared to mock inoculated pigs at 5 days post vaccination.

**Genes up**	**LogFC**	**Genes down**	**LogFC**
*ifit2*	2.553934	*pg-2*	−1.64178
*pkia*	2.373655	*c6h19orf33*	−1.64285
*hgf*	2.295012	*zfp36*	−1.66558
*ano5*	2.191831	*bcas4*	−1.67219
*rsad2*	2.176041	*ssc-mir-135-1*	−1.68395
*adam7*	2.075018	*tmem141*	−1.70127
*rab27b*	1.918744	*ddt*	−1.71247
*kiaa1107*	1.892113	*ndufa11*	−1.7917
*loc100520366*	1.842525	*loc100511639*	−1.80764
*tmem178a*	1.816612	*loc100626517*	−1.80975
*ifit1*	1.807552	*atox1*	−1.81446
*epb41l4b*	1.796457	*myadm*	−1.83981
*c1h14orf37*	1.785264	*ccl14*	−1.88256
*tdrd1*	1.777872	*loc100521485*	−1.89069
*rpgrip1*	1.77437	*ssc-mir-125b-2*	−1.92758
*galntl5*	1.714732	*dusp15*	−1.93288
*ifi44*	1.689456	*tmem160*	−1.94606
*ttc39a*	1.678465	*dpm3*	−1.95109
*wdr35*	1.673544	*scgb3a1*	−2.0114
*pln*	1.670908	*ndufb11*	−2.03292

*Top 20 up/down regulated genes shown*.

At day 8 pv, i.e., 3 dpc and thus when viral RNA was detected in the unvaccinated animals (Figure 1E) 138 genes were differentially regulated, with 118 genes significantly less expressed in tonsils of C strain vaccinated pigs compared to mock inoculated pigs (Table 2, Supplementary Table 2). In terms of gene ontology over-representation, an inversion occurred whereby pathways associated with response to virus were now overrepresented in those pigs that were not vaccinated (Supplementary Figure 1).

**Table 2:** Genes differentially regulated in C-Strain vaccinated pigs compared to mock vaccinated pigs, 8 days post vaccination (3 days post challenge) with CSFV.

**Genes up**	**LogFC**	**Genes down**	**LogFC**
*npg1*	2.47	*sprr1a*	−2.65
*pgrmc2*	1.97	*krt78*	−2.68
*lyzl4*	1.83	*lgals7*	−2.71
*tex14*	1.76	*oasl*	−2.73
*pg-2*	1.69	*krt78*	−2.74
*lrg1*	1.58	*gsta1*	−2.86
*loc102158214*	1.54	*cnfn*	−2.88
*pcd1b*	1.50	*sprp*	−2.89
*loc102157463*	1.45	*loc100516001*	−2.91
*npg4*	1.39	*cnfn*	−2.92
*loc100739707*	1.29	*ifit2*	−3.18
*loc100522081*	1.26	*csta*	−3.32
*pcd1e*	1.25	*olfm4*	−3.37
*slc7a8*	1.18	*sprr1a*	−3.43
*loc100514211*	1.17	*spink5*	−3.56
*znf449*	1.11	*tprg1*	−3.63
*c1h9orf116*	1.06	*csta*	−3.80
*kcnip1*	1.02	*krt23*	−3.86
*pr39*	1.01	*pheroc*	−4.05
*tenm3*	1.00	*cldn17*	−4.19

*Top 20 Up/Down regulated genes shown*.

At 10 dpv, thus 5 dpc, 142 genes were differentially regulated, with 127 of these genes expressed less in the vaccinated animals compared to the mock inoculated group (Table 3, Supplementary Table 3). Ontology analysis yielded similar observations as was seen at day 8 with an over-representation of pathways associated with a response to a virus (Supplementary Figure 2) in pigs that were not vaccinated. Notably, among the few genes that were up regulated in the C strain vaccinated pigs was *eomes*, a gene that encodes a transcriptional regulator known to play a role in CD8^+^ T cell differentiation (22). This corresponds with previously published data where CSFV specific CD8^+^ T cells were detected in the same animal cohort described previously (15).

**Table 3:** Genes differentially regulated in C-Strain vaccinated pigs compared to mock vaccinated pigs, 10 days post vaccination (5 days post challenge with CSFV).

**Genes up**	**LogFC**	**Genes down**	**LogFC**
*eomes*	3.35	*loc100157995*	−2.98
*dapl1*	2.94	*ifi44*	−3.14
*il21*	2.83	*ifit5*	−3.19
*gzmk*	2.74	*cd101*	−3.20
*rgs5*	2.74	*loc100511472*	−3.24
*loc100516016*	2.65	*rsad2*	−3.29
*apitd1*	2.57	*oas1*	−3.40
*loc100512025*	2.40	*loc100518694*	−3.46
*pcdh15*	2.34	*loc100525838*	−3.51
*loc100153678*	2.26	*dhx58*	−3.62
*abca8*	2.24	*irg1*	−3.77
*loc100523628*	2.12	*loc100511550*	−3.98
*loc100521080*	1.98	*ube2l6*	−4.15
*cacnb4*	1.90	*fcgr1a*	−4.15
*loc100512149*	1.81	*loc100512690*	−4.23
		*usp18*	−4.28
		*cxcl11*	−4.55
		*oasl*	−4.78
		*slpi*	−4.90
		*ifit2*	−5.72

*Top 20 Up/Down regulated genes shown*.

### Specific Sub-sets of Genes Fluctuate in Response to CSFV Regardless of Strain Virulence

**Figure 3:**
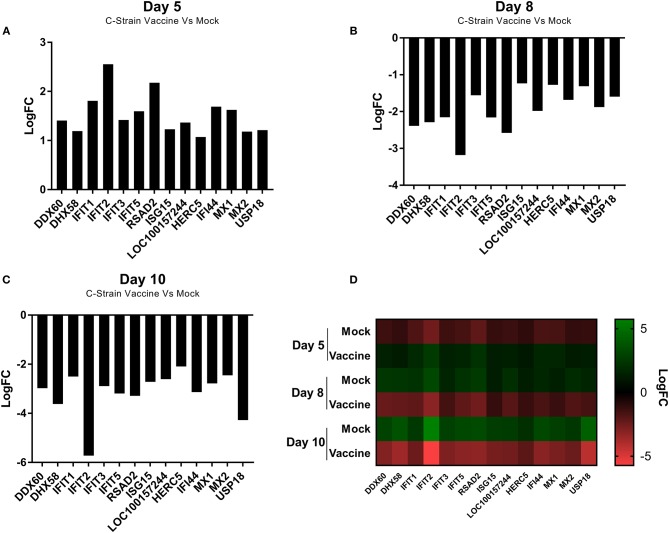
Differential expression of a cohort of genes as identified at each time point. **(A)** Expression of a cohort of 14 genes at day 5 post vaccination, comparing the C Strain vaccinated animals to those that received the mock inoculation. **(B)** Expression of a cohort of 14 genes at day 8 post vaccination (3 dpc) comparing the C Strain vaccinated animals to those that received the mock inoculation. **(C)** Expression of a cohort of 14 genes at day 10 post vaccination (5 dpc), comparing the C Strain vaccinated animals to those that received the mock inoculation. **(D)** Heat map showing the gene expression changes as they occurred over the course of the study. Expression values are from 3 pigs per condition per time point. Significance was determined using a moderated *t*-test *p* < 0.05 considered as significant.

Analysis of all the significantly differentially expressed genes at day 5, 8 and 10 post-vaccination revealed a cohort of genes that were differentially expressed at all of the time points. This suggested that these genes were integral to the response to both the C strain vaccine and the virulent CSFV strain Brescia. These genes were significantly upregulated in C strain vaccinated pigs at 5 dpv (Figure 3A). However, by day 8 and day 10 the expression of these genes in vaccinated animals had partially alleviated suggesting they were no longer induced. Remarkably, this same subset of genes was instead induced significantly in the mock inoculated animals at 8 days (Figure 3B) and 10 days (Figure 3C) post-vaccination (3 and 5 days post-challenge) (Figure 3D). The expression of these genes corresponds with exposure to either strain of the virus and could potentially play a key role in enabling vaccinated pigs to overcome challenge. Indeed, among this cohort are a number of genes coding for antiviral effectors, such as IFIT1, IFIT2, IFIT3, IFIT5 which encode proteins that directly interact with viral RNA preventing the initiation of translation (23–28). As well as MX1 and MX2, proteins that can directly prevent viral ribonucleoprotein complex formation (29–35). The increase in expression of the genes encoding these antiviral effectors, as well as other proteins involved in the innate immune response, such as RSAD2 (Viperin), DDX60 and DHX58, at the time of challenge may play a role in the early protection offered by C strain vaccination.

### The ISG15 Pathway Is Activated in Response to C Strain Vaccination

**Figure 4:**
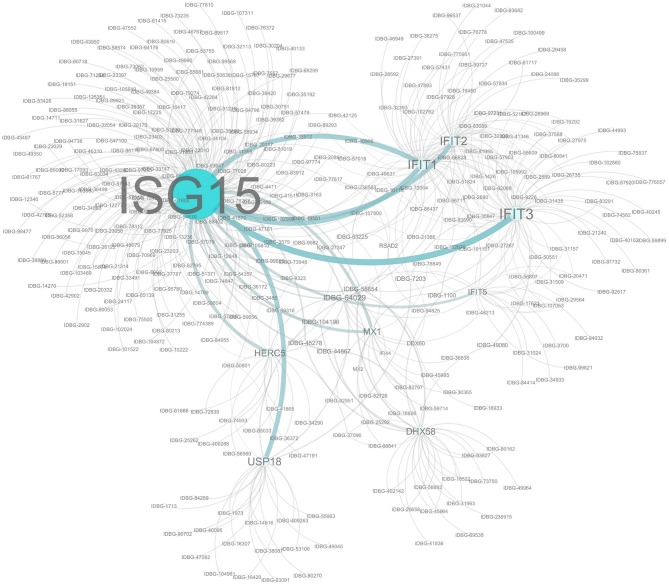
Network analysis of Co-expressed Genes. Network assembled from a cohort of 14 genes significantly differentially regulated at each time point. Network based on interactions defined in the InnateDB. Nodes and label sizes are mapped based on betweenness centrality. Network assembled using Cytoscape 3.2.

The proteins encoded by the subset of genes differentially expressed across all three time points were subjected to an interaction analysis using Cytoscape and pathways from the InnateDB database. This network analysis revealed that many of the proteins within this cohort are capable of directly interacting with at least one other protein in the cohort and also highlighted ISG15 as the best connected node within the network (Figure 4). This is likely to be expected given the nature of ISG15, which functions in a pathway similar to the ubiquitination pathway, in that ISG15 is conjugated to a range of host and non-host proteins modifying their function in a process known as ISGylation (36). Indeed among our common cohort of genes differently expressed at all time points were a number of known ISG15 conjugation targets such as IFIT1-3, IFIT5, DHX58, MX1 (37), as well as other components of the ISG15 pathway including key enzymes HERC5 and USP18, which are directly involved in the ISGylation conjugation and deconjuguation process, respectively (38).

Gene overrepresentation analysis using the Reactome Database identified the Interferon signaling pathway and also identified the ISG15 pathway as being significantly overrepresented across all time points (*p* < 4.04E-09, Supplementary Table 4), albeit in different groups at each time point. It was overrepresented in C strain vaccinated pigs at 5 dpv (*p* < 5.99E-03, Supplementary Table 5), but in mock inoculated pigs at day 8 (*p* < 1.83E-07, Supplementary Table 6) and 10 dpv (*p* < 6.97E-06, Supplementary Table 7).

The early induction of the ISG15 pathway may play a role in the early protection afforded by the C strain vaccination as it ensures that an innate immune response that is producing numerous antiviral effectors (IFIT1, IFIT2, IFIT3, IFIT5, MX1 and MX2) is elevated during this early window.

## Discussion

Since its introduction in the early 1960's the C strain vaccine, has proven remarkably effective and is still the most used vaccine to control CSFV in endemic settings for example in SE Asia (39). It has been shown to stimulate an adaptive cell mediated immune response within 8–10 dpv as previously demonstrated in this animal cohort (15). However, vaccinated pigs are protected already 5 dpv, with partial protection observed even earlier (11, 12). Understanding the molecular mechanisms underpinning this early immunity may aid in the development of more effective, rapid vaccines and in the optimization of vaccines that are currently available. In this study we used a transcriptomic approach to identify a subset of genes that are regulated after both vaccination and challenge and that are linked to a distinct antiviral pathway that is up-regulated during this early protective window.

Type I and III IFNs are known to play a key role in generating a robust host immune response to viral infection. The observed expression of interferon stimulated genes (ISG) with vaccination or challenge had been expected since it is known that CSFV is a strong inducer of type I IFNs, with the main cellular source identified as plasmacytoid dendritic cells (5). C strain localizes specifically in the tonsils which are also the initial site of virus replication during natural CSFV infections, thus the presence of ISGs should be expected first and foremost to be detected locally—but as the infection spreads induction may, in particular, be reflected in other lymphoid tissue (40–42). The interaction of type I IFN with CSFV has been extensively studied, not least as CSFV also exhibits ways to suppress type I IFN (43). While the interferon responses to viral infections are well studied and comprise of over 300 ISGs (44) the precise mechanisms through which these signaling cascades mediate the numerous antiviral responses are yet to be fully elucidated, particularly in the case of CSFV. We focussed here on a set of genes that was significantly regulated across both studied conditions (vaccination and challenge) and was significantly regulated at all time points studied.

Expression of the ISG15 gene has previously been shown to be induced in response to virulent strains of CSFV *in vitro* (45, 46). However, this is the first study to demonstrate induction of the ISG15 pathway in response to vaccination with C strain *in vivo* and specifically that this induction occurs in the tonsil, the primary site of CSFV replication. Further studies can now target genes both up- and down-stream of ISG15 to elucidate in more detail (and possibly with improved sensitivity), which pathways are regulated in response to C strain vaccination. It is noteworthy that ISG15 is not only upregulated through the action of type I IFN, but also IFN-λ and has been shown to be induced via PU.1 a gene expressed in various myeloid cells, including DC (47, 48). Importantly, the ISG15 pathway was up-regulated in C strain vaccinated animals during the window in which a protective immune response exists and the adaptive immunity develops. Although the C strain vaccine was given intramuscularly it is well-established that CSFV has a tropism for tonsil tissue which is the primary site of replication of CSFV (4). The elevation of the ISG15 pathway in this specific tissue is ideally placed to prevent challenge by the most likely natural route of infection.

ISG15 plays a central role in mediating IFN-induced host antiviral responses. ISG15 is a 15 kDa protein that is covalently attached to its target proteins via the action of a group of 3 enzymes (UBE1L, UBCH8, and HERC5), which are also induced in response to type I IFN. This pathway is similar to that of ubiquitination, however unlike ubiquitination, conjugation of ISG15 to host target proteins does not prime them for degradation but instead stabilizes or activates them. Over 150 host ISG15 conjugation targets have been identified thus far (37) Among this cohort of ISGylation targets are some anti-viral proteins whose mRNA has been identified as differentially regulated through our analysis such as IFIT1, IFIT2, IFIT3, IFIT5 MX1, and MX2. These proteins target a number of different aspects of the viral replication cycle such as RNA translation and virion assembly (24–26, 29, 30, 32). Some of the proteins such as MX1, have direct antiviral activity against CSFV (33, 34), other proteins are known to be active against other *Flaviviruses*, such as IFIT2 which restricts growth of West Nile virus (26). Moreover, the free unconjugated form of the ISG15 has antiviral activity and can protect mice against another RNA (Toga-) virus, the Chikungunya virus infection (49).

Conjugation of ISG15 to viral proteins results in their loss of function and the evolutionary importance of this pathway in controlling viral infection is demonstrated by the emerging number of viral proteins that have evolved to disrupt this pathway. For example, the NS1 protein of influenza A and B viruses inhibits ISG15 conjugation (36, 50–52) and NSP2 of porcine reproductive and respiratory syndrome virus, another important pig pathogen, inactivates ISG15 (53).

Further to those proteins directly linked with the ISG15 pathway, we also saw the upregulation of a number of other ISGs. These included IFI44 which is known to have antiviral activity although the precise mechanism of action remains to be characterized (54) and RSAD2 (Viperin) which inhibits many DNA and RNA viruses, including CSFV through interaction with the E2 structural protein (55). Importantly, RSAD2 has also been implicated in DC maturation and CD4 T cell activation (56, 57) and may thus be one of the genes that links the innate and adaptive immune system. One porcine gene LOC100157244 was differentially regulated that has not previously been characterized but is predicted to be an ATP-dependent RNA helicase similar to DDX60. This protein may be a novel component of the pig host's immune response to viral infection and future work needs to focus on characterizing this gene, as well as establishing if some of the other genes upregulated that have not yet been directly related to the ISG15 pathway could represent as yet uncharacterised ISG15 conjugation targets.

**Figure 5:**
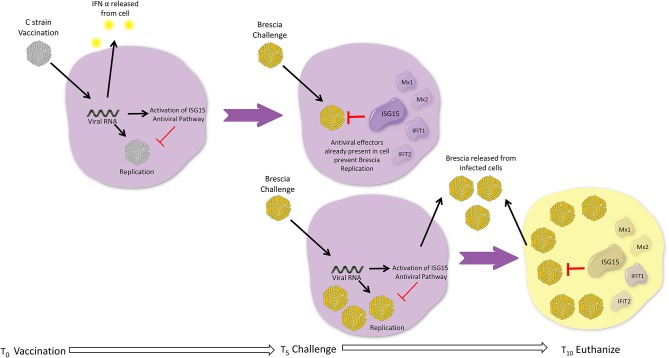
Head Start Immunity Model. Upon vaccination with C strain, the induction of interferon results in induction of ISGs, including the ISG15 antiviral pathway resulting in the induction and activation via ISGylation of a wide variety of antiviral effectors. These antiviral effectors accumulate over the 5 days post vaccination, priming the host in an antiviral state and, for example via induction of RSAD2, instigating the adaptive immune response. If during this window a virulent strain of CSFV attempts to infect the host, the multitude of antiviral effectors are already present within the cell and can immediately prevent the replication of the virus and ultimately assist in preventing the establishment of infection. Without prior vaccination, replication of a virulent strain of CFSV is allowed as although the antiviral effectors of IFN and ISG15 pathways are induced by the virulent virus these cannot keep pace with the replication rate of virulent strains of CSFV and thus are not able to sufficiently control viral replication before adaptive responses can be activated, leading to the onset of clinical disease.

The role of IFN I in CSFV infection has been discussed (5) and it is proposed that the type I IFNs contribute to the pathology of haemorrhagic fever. However, it is well-known that IFN I induce anti-viral effects in cells that have been treated before infection, so that ISGs can be induced, and that a single dose IFN I does not induce a long lasting anti-CSFV effect (58). In light of our analysis, we propose a model whereby C strain vaccination is activating a signaling cascade that is giving vaccinated pigs a head start during which a wide range of innate antiviral effectors are produced, which serve to contain viral replication, should exposure to a virulent strain of CSFV take place prior to the onset of adaptive responses. This is possibly achieved through a number of small changes in several pathways, centering around ISG15, rather than the accumulation of single effector molecules. In naïve hosts, a virulent strain of CSFV will replicate faster, as the innate response cannot produce enough antiviral effectors in time to contain the infection (Figure 5). Future experiments will focus on further exploring the ISG15 pathway temporal dynamics and verifying if the increased transcription of these antiviral effectors is associated with changes in the proteome of the cells. While many of the proteins described have been shown to have direct antiviral activity against CSFV, this response is not necessarily specific to CSFV, but since C strain targets the tonsil, which is also the primary site of CFSV replication, it is particularly effective at protecting against CSFV. Should further analysis corroborate these findings the sustained induction of ISG15 may be a crucial step for successful vaccines. However, the up-regulation of the ISG15 pathway in unvaccinated pigs after CSFV Brescia challenge is most likely associated with the failed attempt of the immune system to induce an antiviral response after infection, contributing to clinical disease including leukopenia (5, 36).

## Ethics Statement

All animal work was approved by the Animal and Plant Health Agency (APHA) Animal Welfare and Ethical Review Board, and all procedures were conducted in accordance with the Animals (Scientific Procedures) Act 1986 (United Kingdom) under project license permits PPL 70/6559.

## Author Contributions

HE, SG, and HC contributed to the performance of the animal experiments and generation of microarray data. RM performed bioinformatics analysis and prepared the manuscript. SG, FS, and HC designed the experiments. All authors reviewed the manuscript.

### Conflict of Interest Statement

The authors declare that the research was conducted in the absence of any commercial or financial relationships that could be construed as a potential conflict of interest.
