# Supplementary material for: Head Start Immunity: Characterizing the Early Protection of C Strain Vaccine Against Subsequent Classical Swine Fever Virus Infection
Source: Front Immunol. 2019 Jul 23;10:1584. doi: 10.3389/fimmu.2019.01584 (PMC6663987; doi:10.3389/fimmu.2019.01584)
Supplement: Supplementary file 4 [file Table_4.pdf]

Supplementary Table 4: Pathways overrepresented in the cohort of genes differentially expressed at every time point.

Pathway (Reactome)	Fold Enrichment	P Value
Interferon alpha/beta signaling (R-HSA-909733)	> 100	2.11E-14
Antiviral mechanism by IFN-stimulated genes (R-HSA-1169410)	> 100	4.04E-09
ISG15 antiviral mechanism (R-HSA-1169408)	> 100	4.04E-09
Interferon Signaling (R-HSA-913531)	75.87	5.38E-13
RIG-I/MDA5 mediated induction of IFN-alpha/beta pathways (R-HSA-168928)	60.7	2.71E-02
Cytokine Signaling in Immune system (R-HSA-1280215)	24.04	1.56E-08
Immune System (R-HSA-168256)	10.15	2.55E-06
Unclassified (UNCLASSIFIED)	0.41	0.00E+00
