# Supplementary material for: Head Start Immunity: Characterizing the Early Protection of C Strain Vaccine Against Subsequent Classical Swine Fever Virus Infection
Source: Front Immunol. 2019 Jul 23;10:1584. doi: 10.3389/fimmu.2019.01584 (PMC6663987; doi:10.3389/fimmu.2019.01584)
Supplement: Supplementary file 5 [file Table_5.pdf]

Supplementary Table 5: Pathways overrepresented at day 5 post vaccination.

Pathway (Reactome)	Fold Enrichment	P Value
Interferon alpha/beta signaling (R-HSA-909733)	9.16	3.94E-04
Antiviral mechanism by IFN-stimulated genes (R-HSA-1169410)	7.77	5.99E-03
ISG15 antiviral mechanism (R-HSA-1169408)	7.77	5.99E-03
Interferon Signaling (R-HSA-913531)	4.72	1.96E-03
Unclassified (UNCLASSIFIED)	0.86	0.00E+00
