# Supplementary material for: Head Start Immunity: Characterizing the Early Protection of C Strain Vaccine Against Subsequent Classical Swine Fever Virus Infection
Source: Front Immunol. 2019 Jul 23;10:1584. doi: 10.3389/fimmu.2019.01584 (PMC6663987; doi:10.3389/fimmu.2019.01584)
Supplement: Supplementary file 6 [file Table_6.pdf]

Supplementary Table 6: Pathways overrepresented at day 8 post vaccination.

Pathway (Reactome)	Fold Enrichment	P Value
Interferon alpha/beta signaling (R-HSA-909733)	33.72	8.75E-11
Negative regulators of RIG-I/MDA5 signaling (R-HSA-936440)	29.75	1.57E-03
Antiviral mechanism by IFN-stimulated genes (R-HSA-1169410)	26.01	1.83E-07
ISG15 antiviral mechanism (R-HSA-1169408)	26.01	1.83E-07
Interferon Signaling (R-HSA-913531)	16.86	5.09E-12
RIG-I/MDA5 mediated induction of IFN-alpha/beta pathways (R-HSA-168928)	15.17	5.91E-03
Cytokine Signaling in Immune system (R-HSA-1280215)	6.34	2.72E-07
Immune System (R-HSA-168256)	3.3	8.19E-05
Unclassified (UNCLASSIFIED)	0.88	0.00E+00
