# Supplementary material for: Head Start Immunity: Characterizing the Early Protection of C Strain Vaccine Against Subsequent Classical Swine Fever Virus Infection
Source: Front Immunol. 2019 Jul 23;10:1584. doi: 10.3389/fimmu.2019.01584 (PMC6663987; doi:10.3389/fimmu.2019.01584)
Supplement: Supplementary file 7 [file Table_7.pdf]

Supplementary Table 7: Pathways overrepresented at day 10 post vaccination.

Pathway (Reactome)	Fold Enrichment	P Value
Interferon alpha/beta signaling (R-HSA-909733)	35.42	3.40E-12
Antiviral mechanism by IFN-stimulated genes (R-HSA-1169410)	22.27	6.97E-06
ISG15 antiviral mechanism (R-HSA-1169408)	22.27	6.97E-06
Interferon Signaling (R-HSA-913531)	16.24	9.44E-12
RIG-I/MDA5 mediated induction of IFN-alpha/beta pathways (R-HSA-168928)	14.61	7.36E-03
Cytokine Signaling in Immune system (R-HSA-1280215)	6.75	8.62E-09
Immune System (R-HSA-168256)	4.28	1.17E-10
Unclassified (UNCLASSIFIED)	0.7	0.00E+00
Interferon alpha/beta signaling (R-HSA-909733)	35.42	3.40E-12
Antiviral mechanism by IFN-stimulated genes (R-HSA-1169410)	22.27	6.97E-06
