# Supplementary figures and images for: Head Start Immunity: Characterizing the Early Protection of C Strain Vaccine Against Subsequent Classical Swine Fever Virus Infection
Source: Front Immunol. 2019 Jul 23;10:1584. doi: 10.3389/fimmu.2019.01584 (PMC6663987; doi:10.3389/fimmu.2019.01584)

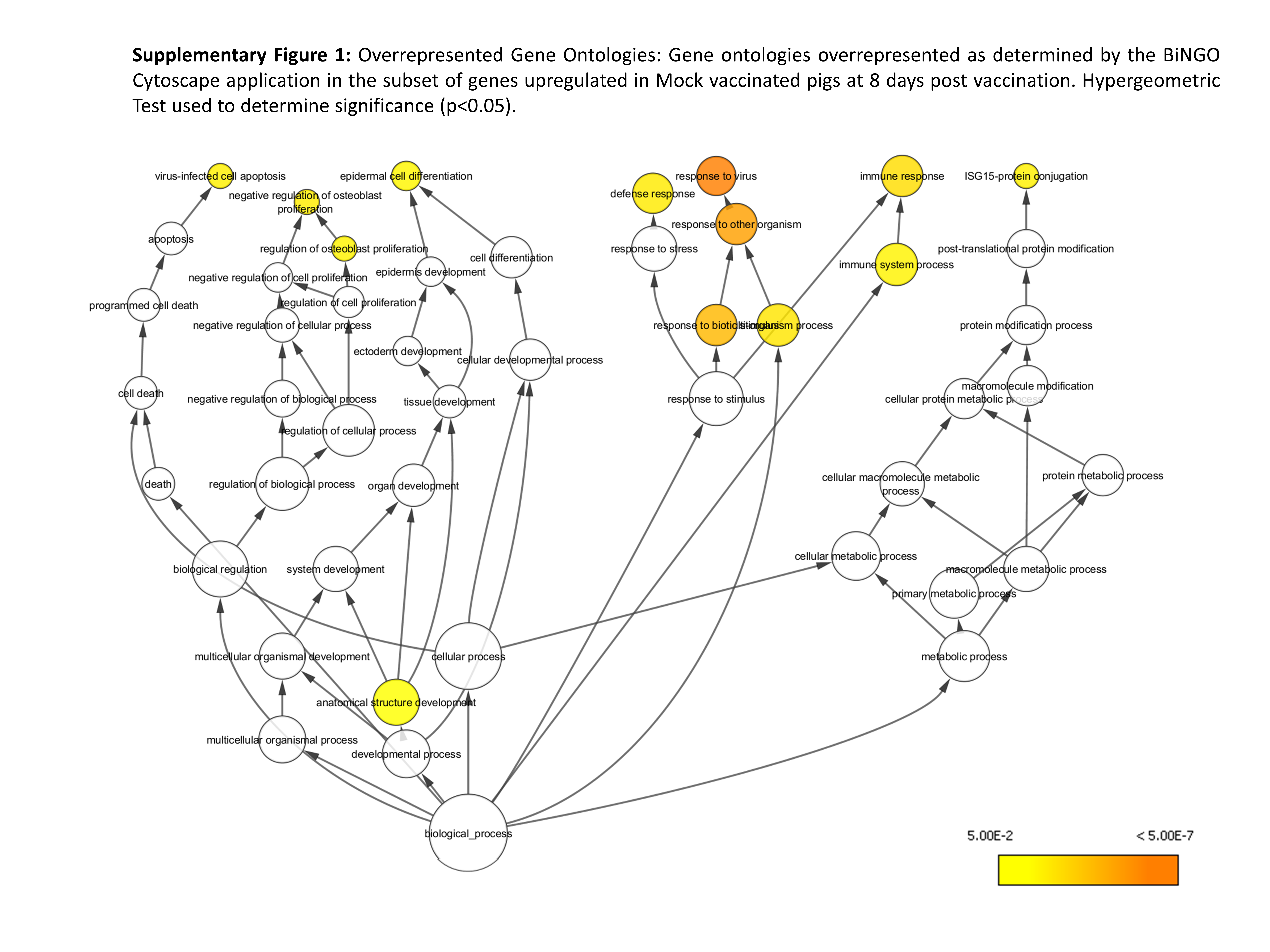

Supplement: Supplementary file 8 [file Image_1.TIF]

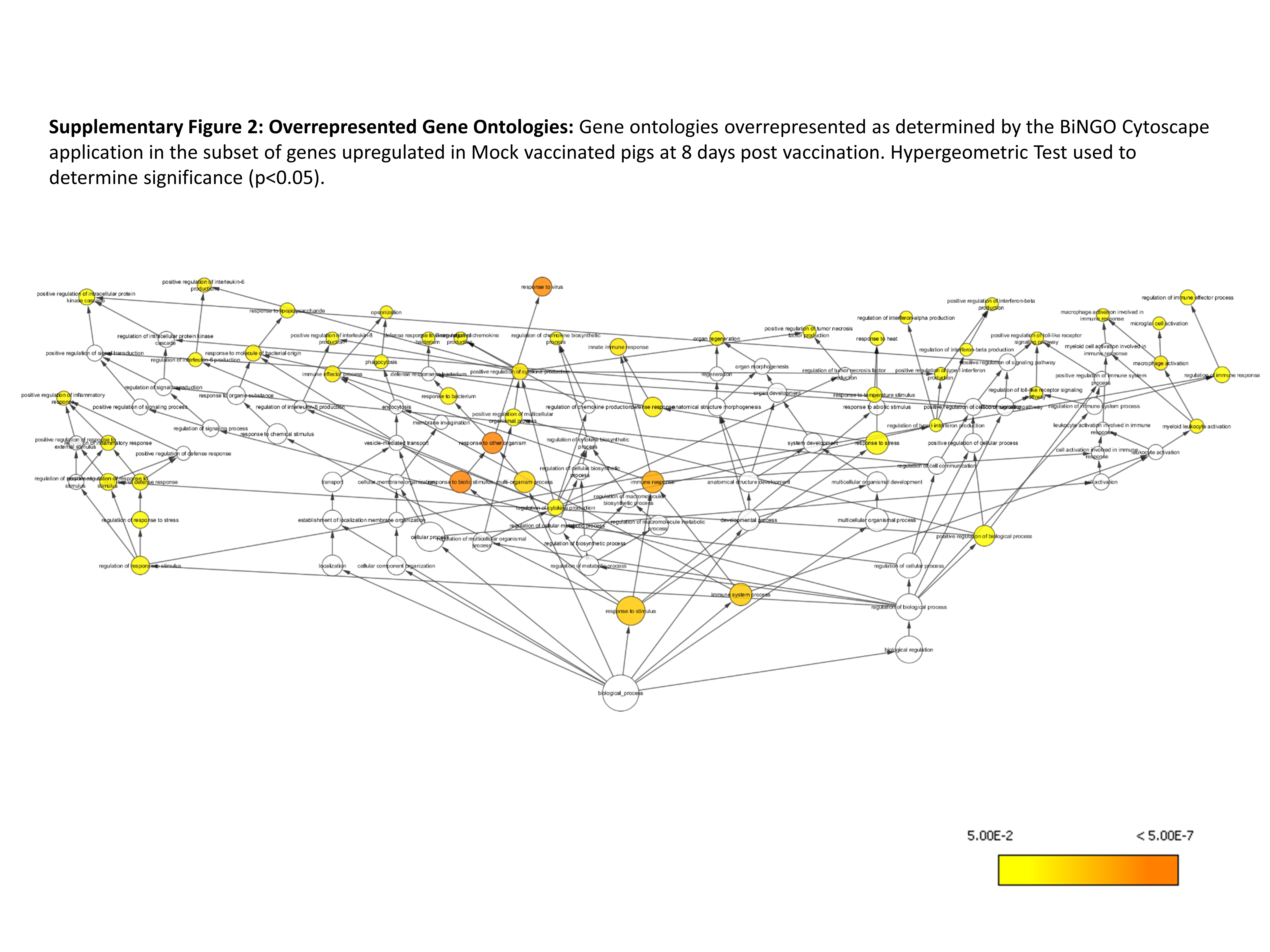

Supplement: Supplementary file 9 [file Image_2.TIF]
